# Associations between pressure pain threshold in the neck and postural control in patients with dizziness or neck pain – a cross-sectional study

**DOI:** 10.1186/s12891-019-2922-4

**Published:** 2019-11-10

**Authors:** Mari K. Knapstad, Frederik K. Goplen, Tove Ask, Jan S. Skouen, Stein Helge G. Nordahl

**Affiliations:** 1 Norwegian National Advisory Unit on Vestibular Disorders, Department of Otorhinolaryngology & Head and Neck Surgery, Haukeland University Hospital, Jonas Lies vei 65, 5021 Bergen, Norway; 2 Department of Clinical Medicine, University of Bergen, Bergen, Norway; 3 Department of Physical Therapy, Haukeland University Hospital, Bergen, Norway; 4 Department of Physical Medicine and Rehabilitation, The Outpatient Spine Clinic, Haukeland University Hospital, Bergen, Norway; 5 Department of Global Public Health and Primary Care, University of Bergen, Bergen, Norway

**Keywords:** Posturography, Neck pain, Dizziness

## Abstract

**Background:**

It is theorized that neck pain may cause reduced postural control due to the known physiological connection between the receptors in the cervical spine and the vestibular system. The purpose of this study was to examine whether the pressure pain threshold in the neck is associated with postural sway in patients with dizziness or neck pain.

**Methods:**

Consecutive patients with dizziness (*n* = 243) and neck pain (*n* = 129) were recruited from an otorhinolaryngological department and an outpatient spine clinic, respectively. All subjects underwent static posturography. Pressure pain thresholds were measured at four standardized points in the neck, and generalized pain was assessed using the American College of Rheumatology tender points. The relationship between postural sway and pressure pain threshold was analyzed by linear regression, and the covariates included age, sex, and generalized pain.

**Results:**

In the dizzy group, there was a small, inverse relationship between pressure pain thresholds and sway area with eyes closed, after adjusting for age, sex, and generalized pain (bare platform; lower neck, *p* = 0.002, *R*^*2*^ = 0.068; upper neck, *p* = 0.038, *R*^*2*^ *=* 0.047; foam rubber mat; lower neck, *p* = 0.014, *R*^*2*^ = 0.085). The same inverse relationship was found between pressure pain thresholds in the neck and the Romberg ratio on a bare platform after adjusting for age, sex and generalized pain (upper neck, *p* = 0.15, *R*^*2*^ = 0.053; lower neck, *p* = 0.002, R^2^ = 0.069). Neither of these relationships were present in the neck pain group.

**Conclusion:**

Our findings indicate that the pressure pain threshold in the neck is associated with postural sway in patients suffering from dizziness after adjusting for age, sex, and generalized pain, but only with closed eyes. The association was small and should be interpreted with caution.

**Trial registration:**

Trial registration: Clinicaltrial.gov NCT03531619. Retrospectively registered 22 May 2018.

## Background

Postural control is a complex system [1] and to maintain control, the body requires input from the vestibular, visual, and somatosensory systems. As part of the somatosensory system, the proprioceptive system in the cervical spine is vital for fine tuning orientation and balance [2]. This proprioceptive system consists of the deep cervical muscles, particularly the segmental muscles of the upper spine – with an abundance of muscle spindles – in addition to mechanoreceptors from joints and tendons. This system is important for both the stability and the mobility of the different regions in the neck. The cervical receptors provide afferent information to the central nervous system on the orientation of the head with respect to the rest of the body via modulation of vestibular and visual afferent information [3]. Integration of symmetrical afferent input from the cervical, vestibular, and visual systems in the vestibular nuclei complex is vital for normal head perception and postural control, and for providing responses resulting in precise motor commands to the eyes and body [3, 4]. Thus, it is theorized that an asymmetry or disturbance of inputs from cervical receptors might lead to a feeling of imbalance or dizziness [3, 4]. The mechanism by which reduced cervical proprioception might lead to sensory disturbances and reduced postural control is still uncertain and disputed, even though the confluence of vestibular and cervical afferents in the brain is well known [5]. It has, however, been proposed that pain, either as a primary or secondary event, may lead to altered sensitivity of the muscle spindles and mechanoreceptors due to ischemic or inflammatory events [6]. Further, pain may cause maladaptive strategies and change the neck muscle coordination and reduce the specificity of neck muscle activation, for instance, through reduced activation of the deep segmental muscles and increased activation of the superficial muscles [7]. Pain may also alter the cortical representation and modulation of the cervical afferent input [8]. The relationship between altered neck proprioception and pain has been found in healthy subjects receiving injections to induce neck pain [9], and animal studies have shown that local injections, nerve blockades, and dissection of neck muscle in the upper cervical region, lead to decreased balance, coordination, ataxia, and even nystagmus [10–12]. Lastly, both patients with chronic neck pain and whiplash-related disorders have been found to have reduced postural control [13], and the same has been found in patients with dizziness of suspected cervical origin [14–18]. The relationship has previously been mostly studied in patients with neck pain; however, it is not established whether the degree of neck pain is associated with the degree of postural control. It is also not known if neck pain influences postural control in dizzy patients as many patients with dizziness suffer from neck pain [19, 20]. Exploring this relationship in both patients with dizziness and patients with neck pain may provide information on how the degree of neck pain influences postural control in two patient groups known to have altered balance.

Self-reported pain intensity has been the most common approach to pain measurement. While self-reported pain is indeed important, it is mediated by biopsychosocial aspects [21] that can make it difficult to interpret. The pressure pain threshold (PPT) is a tool of both self-report but additionally a more objective technique [22] that is used to quantify mechanical pain sensitivity [23, 24]. It is defined as the minimal amount of pressure that first becomes on of pain [25].

The main aim of this study is to examine whether there is an association between PPT and postural sway in patients with dizziness and in patients with neck pain. As patients with pain syndromes, such as Fibromyalgia, have been shown to have reduced balance [26, 27] and patients rarely have isolated neck pain as it is usually a part of a wider pain pattern [28], we wanted to adjust for generalized pain. Finally, we wanted to examine the upper and lower regions of the cervical spine separately due to their differences in mechanical properties and distribution of mechanoreceptors.

## Methods

### Design and setting

We conducted a prospective cross-sectional study of consecutive outpatients examined at two clinical centers at a university hospital in Norway. The first center was an ear-nose-throat (ENT) clinic that receives referrals from general practitioners and specialists, both nationally and locally, concerning dizziness of suspected vestibular origin. The second center was an outpatient spine clinic that admits patients from primary care physicians concerning long-lasting musculoskeletal pain either causing or threatening to cause work disability.

### Participants

**Fig. 1:**
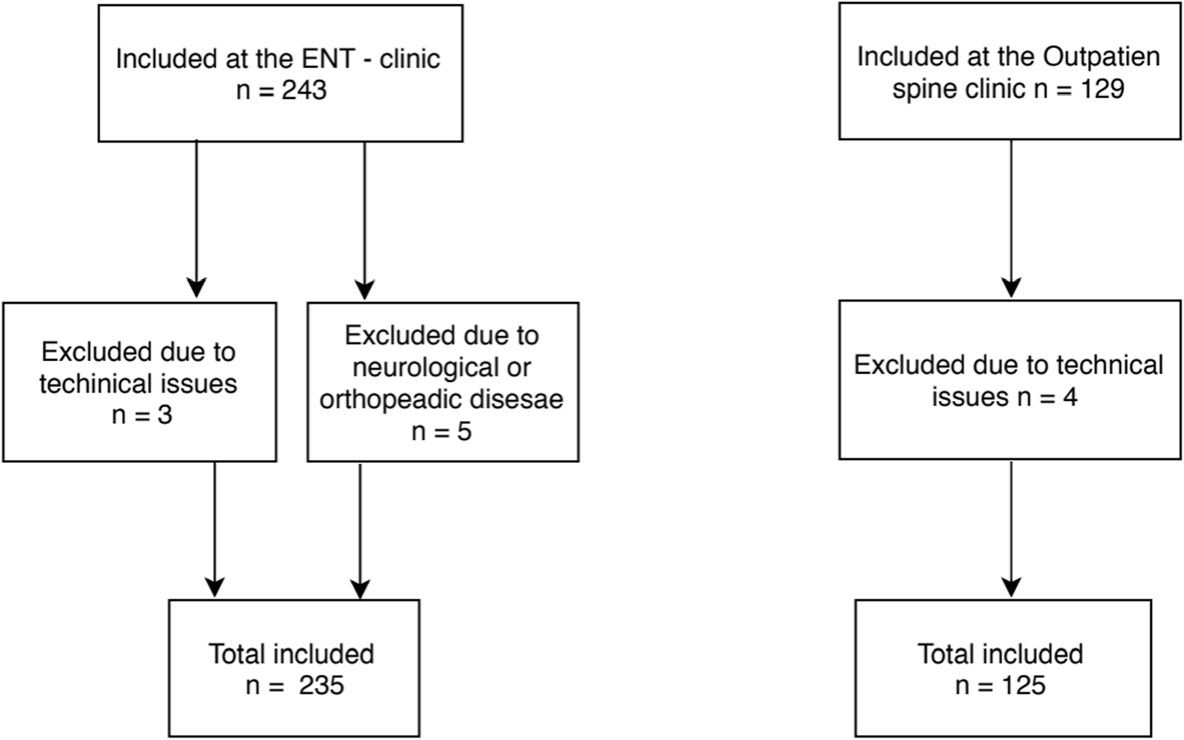
Recruitment process

During a one-year period (2017–2018), we included consecutive patients examined in both clinics. The recruitment is illustrated in Fig. 1. Patients with dizziness as their primary complaint (*n* = 243) were recruited from the ENT clinic. The ENT clinic also receives tertiary referrals nationally and is a quaternary referral center for vestibular schwannomas and for divers suffering from vestibular problems. As we wanted the study population to be representative of secondary referrals, persons having the latter conditions were not invited to participate (based on medical records) and only locally referred patients from western Norway were included. They were diagnosed by an otorhinolaryngologist, and the examination included pure-tone audiometry, dynamic posturography, videonystagmography with measurements of ocular smooth pursuit, saccades and bithermal caloric tests, a standard ENT examination including otomicroscopy, examination of cranial nerves and cerebellar function as well as clinical tests of postural sway, gait, and nystagmus. In addition, hospitalized patients with acute vertigo were also excluded.

Patients with long-lasting (> 3 months) neck pain (*n* = 129) as their primary complaint were recruited from the outpatient spine clinic where they were examined by a multidisciplinary team and diagnosed by a physician. In both groups, the participants had to be between 18 and 67 years old. Exclusion criteria included language barriers associated with filling in patient questionnaires and neurological or orthopedic disorders known to interfere with postural control (these were excluded prior to invitation to participate based on medical records). The project was approved by the Regional Committee for Health and Medical Research Ethics of South-Eastern Norway (REK 2017/783). The participants signed a written consent prior to testing.

### Pressure pain threshold

Neck PPT were used to quantify the mechanical pain sensitivity of the cervical region using a pressure algometer. This threshold has previously been found to predict shoulder/neck pain [29] and to correlate with other measures of neck pain [30]. The PPT was measured in all subjects in the prone position by trained physiotherapists. A Wagner FDX-25 digital force gage (Wagner Instruments, Greenwich, CT) with a linear response of 0–1300 kilopascals (kPa) and a 1 cm^2^ round rubber tip was used to apply pressure to the upper four standardized and defined American College of Rheumatology (ACR) tender points [31]: bilaterally suboccipital and 2 cm lateral to the spinous process of the axis (upper neck) and bilaterally at the anterior aspects of the intertransverse space at C5–C7 (lower neck). The algometer has been shown to be a reliable tool on these sites in dizzy patients with intraclass correlation values of 0.82–0.90 on intrarater reliability and 0.85–0.91 on test–retest reliability. The minimal detectable change showed values from 44.5 kPa – 86.1 kPa on intrarater reliability and 77.7 kPa – 88.2 kPa on test–retest reliability [30]. Prior to the study, the examiners practiced applying pressure at a rate of approximately 50 kPa/s. The digital force gage maintained its peak value, and the examiner was blinded to the display while applying pressure. The patient was told to immediately state when the pressure sensation changed into a pain sensation, at which time the pressure was stopped and the score was noted. A lower score indicated a greater degree of pain sensitivity. Three measurements were recorded at each site, starting on the left at the suboccipital site and ending on the right on the intertransverse space at C5–C6. As the last two measurements have been found to have the highest reliability [30], we used the mean of those measures for further analysis.

### Generalized pain

Pressure testing at the 18 ACR tender points was used to measure the level of generalized pain. The test assesses nine defined points on each side of the body as illustrated in Wolfe et al. (1990) [31]. The tester gradually administered increasing pressure to each point, stopping at approximately 4 kg pressure. The patient was told to say “yes” if they experienced pain or “no” if they experienced only discomfort at each point after pressure was applied. The number of tender points (0–18) was used in further analysis.

### Postural control

Postural control was evaluated by static posturography using a commercially available force platform (Synapsys, Marseille, France). The center of pressure under the feet was sampled at a rate of 100 Hz. The evaluated parameter was the sway area in mm^2^ described by the center of pressure during each test lasting 2 × 20 s. The patients were instructed to stand quietly on the force platform with their arms hanging freely along their body and their feet aligned with markings corresponding to their foot size. To evaluate the different contributions of proprioceptive and visual inputs, the patients were tested under four different conditions: eyes open or eyes closed while standing on the bare platform and eyes open or closed while standing on a foam rubber mat placed on top of the platform. Additionally, as an indicator of the proprioceptive contribution to postural stability the Romberg ratio [32] was calculated as the sway area with eyes closed divided by the sway area with eyes open with and without the foam rubber on the platform. A higher ratio, and thus greater difference between eyes closed and eyes open, indicates greater proprioceptive deficit as they rely more on vision to maintain postural control.

### Procedure sequence

A study nurse at each clinic recruited the patients the same day as their appointment at their respective clinic. Four experienced physiotherapists conducted subsequent testing on the day of their appointment. To ensure consistent examination techniques, the examiners had two practice sessions before the study and one more after 5 months. Before testing, the participants filled in medical chart data such as age, sex, and symptom characteristics. The examination was carried out in the following sequence: PPT, ACR tender points, and posturography. At the ENT clinic, the patients were examined before or after their physician appointment, and at the outpatient spine clinic the patients were tested as a part of the physiotherapy examination and after they were examined by a physician.

### Statistical analysis

Linear regression was used to estimate the relationship between postural sway (sway area and Romberg ratio) and PPT after adjusting for age, sex, and generalized pain (number of ACR tender points). Sway area was used as the dependent variable and PPT as the predictor variable. Three regression models were generated, including the unadjusted model (Model 1), the age and sex-adjusted model (Model 2), and the age, sex, and generalized pain-adjusted model (Model 3). The alpha level was set to 0.05. Descriptive statistics included means and standard deviations for normally distributed data or median and interquartile range for skewed data. Categorical data were presented as percentages. The sample size was estimated based on recommendations by Green [33], which state that for a power of 0.8 the minimum sample size should be 104 + *m* where *m* is the number of predictors; thus, resulting in a sample size of at least 105 patients for each regression analysis. Sway area and Romberg ratio were positively skewed and were logarithmically transformed prior to regression analysis. PPT in the upper and lower neck was highly correlated and thus assessed in separate analyses to avoid multicollinearity. To facilitate interpretation of the coefficients, they were back transformed after analysis. Statistical analysis was performed using Stata® version 15 (StataCorp, Texas, USA).

## Results

This study included 243 patients from the ENT clinic with dizziness and 129 patients from the spine clinic with neck pain. Due to technical issues, three patients from the ENT clinic and four patients from the spine clinic had to be excluded because of missing posturography data. In addition, five patients from the ENT clinic were excluded due to neurological or orthopedic disorders that were not uncovered prior to participation in the study. Diagnoses of the different populations are shown in Additional file 1: Table S1. In the neck pain group, only 17% (21 patients) reported a neck injury as the trigger for their neck pain. Descriptive statistics are given in Table 1.

**Table 1:** Descriptive data on postural control, neck PPT, and generalized pain

Variable	Dizziness group (*n* = 235)	Neck pain group (*n* = 125)
Age	45.7 ± 12	41 ± 11
Sex (female) (%)	73.5%	79.2%
Duration dizziness, months^a^	12 (6–38)	
Duration neck pain, months^a^		14 (5–89)
Concurrent complaints (%)	53%	45%
Posturography^a^, sway area, mm^2^		
-Eyes open; bare platform	226 (148–419)	144 (93–212)
-Eyes closed; bare platform	403 (243–904)	213 (124–328)
-Eyes open; foam mat	544 (346–887)	277 (194–368)
-Eyes closed; foam mat	1662 (1019–2956)	639 (432–1028)
Romberg ratio^a,c^		
-Bare platform	1.85 (1.13–2.74)	1.49 (1.07–1.98)
-Foam mat	3.12 (2.19–4.39)	2.26 (1.71–3.14)
PPT, kPa^b^		
-Upper neck	216.7 ± 112.8	219.3 ± 115.8
-Lower neck	184.0 ± 86.4	192.8 ± 96.6
Generalized pain (ACR count)	9.2 ± 5.9	7.7 ± 4.9

^a^Reported as median and interquartile range, ^b^Reported as mean and standard deviation, ^c^Romberg ratio = sway area with eyes closed divided by sway area with eyes open. *PPT* Pressure pain threshold, *ACR* American College of Rheumatology tender points, *n* sample size. *Concurrent complaints* presence of both dizziness and neck pain the last 14 days

PPT was not significantly associated with postural sway with eyes open with or without the foam mat in any of the groups. After adjusting for age, sex, and generalized pain, there was an inverse relationship between PPT and sway area in both the eyes closed conditions (with and without foam) in the lower neck in the dizziness group. An increase of 10 kPa was associated with a 3.1% reduction of sway in the eyes closed condition (95% confidence interval [CI], − 5.0 to − 1.1%, *p* = 0.002) and a 1.8% reduction of sway in the eyes closed on foam condition (95% confidence interval [CI], − 3.3 to − 0.4%, *p* = 0.014). In the upper neck, there was an inverse relationship between PPT and sway area in the third model, when standing with eyes closed on bare platform and an increase of 10 kPa was associated with a 1.6% reduction of sway in the eyes closed condition (95% confidence interval [CI], − 3.1 to − 0.1%, *p* = 0.038). In the patients with neck pain, PPT was not associated with postural sway in any of the models (Table 2).

**Table 2:** Linear regression analysis between the logarithm of sway area and neck PPT in persons with dizziness (*n* = 234) and in persons with neck pain (*n* = 125)

	PPT Upper Neck			PPT Lower Neck		
Groups	*B*(CI)	*p*	R^2^	*B* (CI)	*p*	R^2^
Eyes open						
Model 1: Unadjusted						
Neck Pain	−.0001 (−.0013 to .0010)	.815	.0004	−.0003 (−.0018 to .0011)	.637	.0020
Dizziness	−.0002 (−.0013 to .0007)	.569	.0014	−.0007 (−.0021 to .0005)	.258	.0055
Model 2: Adjusted for age and						
Neck Pain	−.0005 (−.0018 to .0008)	.5436	.0468	−.0007 (−.0023 to .0008)	.363	.0485
Dizziness	−.0007 (−.0018to .0003)	.181	.0318	−.0014 (−.0028 to <−.0001)	.047	.0409
Model 3: Adjusted for age, sex, and GP						
Neck Pain	−.0003 (−.0017 to .0010)	.625	.0495	−.0005 (−.0022 to .0012)	.548	.0504
Dizziness	−.0004(−.0016 to .0007)	.432	.0357	−.0011(−.0027 to .0093)	.124	.0431
Eyes open on foam						
Model 1: Unadjusted						
Neck Pain	<.0001(−.0009 to .0009)	.977	<.0001	<−.0001 (−.0012 to .0011)	.930	.0001
Dizziness	−.0002 (−.0013 to .0007)	.555	.0015	−.0001 (−.0013 to .0009)	.769	.0004
Model 2: Adjusted for age and sex						
Neck Pain	−.0001 (−.0012 to .0009)	.780	.0492	−.0002 (−.0014 to .0011)	.799	.0491
Dizziness	−.0006 (−.0016 to .0004)	.263	.0295	−.0012 (−.0026 to .0001)	.086	.0367
Model 3: Adjusted for age, sex, and GP						
Neck Pain	.0001 (−.0010 to .0012)	.838	.0602	.0002 (−.0011 to .0016)	.764	.0606
Dizziness	−.0003 (−.0015 to .0009)	.626	.0345	−.0009 (−.0024 to .0006)	.234	.0394
Eyes closed						
Model 1: Unadjusted						
Neck Pain	−.0003 (−.0017 to .0009)	.592	.0023	−.0004 (−.0021 to .0011)	.601	.0022
Dizziness	−.0015 (−.0027 to −.0002)	**.020**	.0230	−.0026 (−.0042 to −.0009)	**.002**	.0408
Model 2: Adjusted for age and sex						
Neck Pain	−.0005 (−.0019 to .0009)	.471	.0786	−.0004 (−.0021 to .0012)	.613	.0766
Dizziness	−.0018 (−.0032 to −.0004)	**.010**	.0460	−.0031 (−.0049 to −.0013)	**.001**	.0682
Model 3: Adjusted for age, sex, and GP						
Neck Pain	−.0004 (−.0019 to .0011)	.592	.0794	−.0002 (−.0021 to .0016)	.781	.0778
Dizziness	−.0016 (−.0031 to <−.0001)	**.038**	.0468	−.0031 (−.0050 to −.0011)	**.002**	.0681
Eyes closed on foam						
Model 1: Unadjusted						
Neck Pain	−.0001 (−.0012 to .0010)	.837	.0003	−.0002 (−.0015 to .0012)	.792	.0006
Dizziness	−.0008 (−.0017 to .0002)	.131	.0098	−.0014 (−.0027 to −.0002)	**.028**	.0206
Model 2: Adjusted for age and sex						
Neck Pain	−.0008 (−.0019 to .0004)	.190	.1254	−.0008 (−.0022 to .0006)	.243	.1228
Dizziness	−.0011 (−.0022 to −.0002)	**.024**	.0659	−.0021 (−.0034 to −.0008)	**.002**	.0837
Model 3: Adjusted for age, sex, and GP						
Neck Pain	−.0003 (−.0015 to .0009)	.638	.1517	−.0003 (−.0017 to .0014)	.841	.1504
Dizziness	−.0009 (−.0020 to .0003)	.134	.0700	−.0018 (−.0033 to −.0004)	**.014**	.0852

*PPT* Pressure pain threshold, *R*^*2*^ explained R-squared, *p p*-value, *CI* confidence interval, *B* regression coefficient, *n* sample size, *GP* generalized pain (number of ACR tender points)

Figures in bold indicate significant *p*-value

Regression analysis adjusted for age, sex, and generalized pain found an inverse relationship between PPT and Romberg ratio in both the upper and lower neck on the bare platform in the dizziness group. A 10 kPa increase in PPT in the upper neck was associated with a 1.1% decrease in Romberg ratio (95% confidence interval [CI], − 2.0 to − 0.2%, *p* = 0.015) and a 1.8% decrease in PPT in the lower neck (95% confidence interval [CI], − 3.0 to − 0.7%, *p* = 0.002). On foam rubber, the PPT was only associated with the Romberg ratio in the age and sex-adjusted model. No relationship was found in the neck pain group in either of the conditions (Table 3).

**Table 3:** Linear regression analysis between the logarithm of the Romberg ratio and neck PPT in persons with dizziness (*n* = 234) and in patients with neck pain (*n* = 125)

	PPT Upper Neck			PPT Lower Neck		
Groups	*B* (CI)	*p*	R^2^	*B* (CI)	*p*	R^2^
Romberg ratio						
Model 1: Unadjusted						
Neck Pain	−.0002 (−.0011 to .0007)	.620	.0020	<−.0001 (−.0011 to .0009)	.881	.0002
Dizziness	−.0012 (−.0019 to −.0004)	**.002**	.0413	−.0018 (−.0029 to −.0009)	**<.001**	.0571
Model 2: Adjusted for age and sex						
Neck Pain	<−.0001 (−.0009 to .0009)	.989	.0423	.0002 (−.0009 to .0014)	.625	.0442
Dizziness	−.0010 (−.0018 to −.0002)	**.011**	.0519	−.0017 (−.0028 to −.0006)	**.002**	.0667
Model 3: adjusted for age, sex, and GP						
Neck Pain	<−.0001 (−.0011 to .0009)	.894	.0430	.0002 (−.0009 to .0014)	.625	.0442
Dizziness	−.0011 (−.0020 to −.0002)	**.015**	.0533	−.0018 (−.0030 to −.0007)	**.002**	.0687
Romberg ratio on foam						
Model 1: Unadjusted						
Neck Pain	−.0001 (−.0009 to .0007)	.749	.0008	−.0001 (−.0012 to .0008)	.795	.0006
Dizziness	−.0005(−.0011 to .0002)	.143	.0092	−.0007 (−.0015 to .0009)	.082	.0130
Model 2: Adjusted for age and sex						
Neck Pain	−.0006 (−.0015 to .0002)	.154	.0746	−.0006 (−.0016to .0004)	.207	.0712
Dizziness	−.0005 (−.0012 to <.0001)	.082	.0212	−.0009 (−.0017 to <−.0001)	**.039**	.0264
Model 3: Adjusted for age, sex, and GP						
Neck Pain	−.0004 (−.0013 to .0005)	.383	.0845	−.0003 (−.0014 to .0008)	.530	.0817
Dizziness	−.0005 (−.0013 to .0002)	.120	.0206	−.0009 (−.0018 to <.0001)	.057	.0258

*PPT* Pressure pain threshold, *R*^*2*^ explained R-squared, *p* p-value, *CI* confidence interval, *B* regression coefficient, *n* sample size, *GP* generalized pain (number of ACR tender points)

Figures in bold indicate significant *p*-value

## Discussion

This study found an inverse relationship between PPT in the neck, postural sway, and Romberg ratio. The effect of PPT on sway was small and the association was only present in the eyes closed conditions and only in patients examined at the ENT clinic for dizziness. The inverse relationship indicated that a higher PPT (lower pain sensitivity) was associated with better performance (lower sway area and lower Romberg ratio) on the platform, and thus, a lower PPT (higher pain sensitivity) was associated with worse performance (higher sway area and higher Romberg ratio). The associations tended to remain significant after adjustment for age, sex, and generalized pain.

Previous studies have demonstrated impairments of postural control in patients with assumed cervicogenic dizziness [14–18] and in neck pain patients [13]. However, these studies did not analyse the quantitative relationship between the degree of neck pain and postural control, nor did they adjust for generalized pain. Ruhe et al. (2013) found a linear relationship between the numeric pain rating scale and postural sway in patients with non-specific neck pain [34]. However, in theory, PPT might be a more objective surrogate measure of pain than a subjective rating because subjective measures may be more influenced by both physiological and psychosocial factors [35]. PPT cannot directly measure altered proprioception of the neck, but the theory is that pain in the neck region influences the afferent input, and previous studies have supported this [8, 36].

An association between postural sway and PPT was found in patients examined for dizziness at the ENT clinic. This is an interesting finding. Postural control relies on several sensory systems, and a deficit in one of these may be compensated for by the others. In the ENT clinic, approximately 50% were diagnosed with a vestibular problem. A possible explanation for our findings in this group may be that there was a synergistic interaction between neck pathology and vestibular deficit. Neck pain alone may not be sufficient to cause an association between neck pain and postural imbalance. However, 45% in the neck pain group reported dizziness. It may be speculated that dizziness in most of these patients was non-vestibular, possibly related to their neck pain.

After adjusting for age, sex, and generalized pain, the association with PPT in the neck was only present with eyes closed, i.e. when the patients were deprived of visual feedback. In the eyes closed condition, the central nervous system has to rely on accurate vestibular and somatosensory feedback, including important information about head-on-body position from proprioceptive afferents in the neck [37]. This is corroborated by the association between PPT and Romberg ratio. The Romberg ratio is considered to be an indication of visual dependency due to proprioceptive deficit [32], and we found that a reduction in the Romberg ratio (less sway difference between eyes closed and eyes open) was associated with an increase in PPT in both the upper and lower neck. Seemingly, patients with a higher PPT had a smaller ratio between the eyes closed and eyes open conditions. A possible interpretation of our findings is that a lower PPT in the neck is associated with less reliable cervical proprioceptive information and thus higher visual dependency, therefore causing increased sway in the eyes closed condition compared to the eyes open condition. Other sensory deficits could affect the ratio such as degree of vestibular dysfunction. However, such measures do not seem to associate well with postural control [38]. PPT was not associated with postural sway in the eyes open conditions either with or without the foam mat; however, standing steadiness with eyes open is quite robust in patients with vestibular disorders and in those with proprioceptive disorders [39]. Posturography with eyes closed when standing on foam rubber is considered mostly to rely on vestibular function because vision is eliminated and proprioceptive feedback from the feet is unreliable [39]. In this condition, the brain might choose not to rely on proprioceptive information from the neck as well as from the feet. The finding of a relationship between sway area and PPT in this condition, might indicate that neck proprioception still contributes to postural stability when standing on foam rubber. However, it is important to emphasize that PPT had a small explanatory power for both sway area and Romberg ratio, thus interpretation must be done with caution. The coefficients of the association were small with small changes in percentage of sway. Previous studies examining PPT in the neck area found a minimal detectable change ranging from 69 to 113 kPa [30, 40]. Larger differences in PPT would cause a larger percentage of sway. In addition, it is mostly assumed that dizziness with a suspected cervical origin rarely involves true vertigo and is often characterized with more vaguely described dizziness, such as a feeling of unsteadiness, disequilibrium, or light-headedness [4, 41]. In light of this, it is interesting to speculate whether the association found in this study, however small, might influence a patient’s symptoms and a feeling of unsteadiness.

The relationship between sway area and PPT was most consistent in the lower neck. This was somewhat contradictory to the fact that the mechanoreceptors are more concentrated in the upper region of the cervical spine [3]. The PPT was lower in the lower neck region in both the dizziness group and neck pain group compared to the upper region. One explanation might be that the upper region is the most mobile part of the vertebral column, lack of motor control due to pain might cause the lower region of the cervical spine to compensate, and thus increase stiffness or pain sensitivity in the lower cervical spine. Additionally, it is important to note that the PPT was measured at standardized sites, and therefore perhaps not at the sites that patients perceived to be most painful.

This study has several limitations. First, the coefficients of the association were small with small changes in percentage of sway. In addition, the small explanatory power (R^2^) shows that the PPT has small effects on sway. Although a small significant association was found, there is no consensus on normative values for the sway area; thus, making an interpretation of the importance of the percentage change difficult. However, the aim of this paper was merely to determine whether an association exists between PPT and sway area. The relationship between neck pain and reduced postural control is founded on basic research and experimental evidence showing that the activity of primary vestibular neurons is modulated by proprioceptive afferents in the neck [9–12, 42] making it a plausible explanation for our findings. The populations in this study were heterogeneous as we examined the associations in symptom complexes and not specific diagnoses. Persons with traumatic neck pain were underrepresented in the neck pain group. Even though reduced postural control has been linked to neck pain of non-traumatic origin, it might be more common in patients with traumatic origin of neck pain [13]. Patients referred to the clinics with vestibular schwannomas, diving related inner ear trauma, severe neurologic or orthopedic injuries or referred from other parts of the country were excluded based on the medical referral prior to their visit. However, we did not register how many patients were excluded prior to their visit based on referral information. Still, if any patient reported any severe neurological or orthopedic injury after inclusion, they were registered and excluded. Finally, to examine the same association in a control group would have enhanced this study. A strength of the study was the large sample size and the correction for generalized pain, emphasizing the cervical contribution to postural control. Moreover, the measurements of PPT and postural sway were objective and were performed on two unselected patient groups with dizziness and neck pain, i.e. the patients were not selected due to any a priori assumption of a causal link between their neck symptoms and dizziness.

## Conclusion

This study found an inverse relationship between PPT and postural sway. The association was present with eyes closed in patients suffering from dizziness after adjustment for age, sex, and generalized pain (ACR tender points). In addition, the Romberg ratio was associated with PPT. However, altered postural control has a myriad of possible causes and the effect of PPT on sway was small and needs to be corroborated in future studies.

## Supplementary information

**Additional file 1: Table S1.** Frequency of diagnosis of 231 patients referred to the ENT clinic and the 125 patients referred to the outpatient spine clinic.

## Data Availability

The datasets used and/or analyzed during the current study are available from the corresponding author on reasonable request.

## Abbreviations

ACR: American College of Rheumatology
COP: center of pressure
PPT: pressure pain threshold
