# Supplementary material for: Associations between pressure pain threshold in the neck and postural control in patients with dizziness or neck pain – a cross-sectional study
Source: BMC Musculoskelet Disord. 2019 Nov 10;20:528. doi: 10.1186/s12891-019-2922-4 (PMC6844061; doi:10.1186/s12891-019-2922-4)
Supplement: Supplementary file 1 — Additional file 1: Table S1. Frequency of diagnosis of 231 patients referred to the ENT clinic and the 125 patients referred to the outpatient spine clinic. [file 12891_2019_2922_MOESM1_ESM.docx]

Additional file 1: **Table S1.** Frequency of diagnosis of 231 patients referred to the ENT clinic and the 125 patients referred to the outpatient spine clinic

| Diagnosis at the ENT clinic | n | % |
| --- | --- | --- |
| **Peripheral Vestibular** | 108 | 47 |
| BPPV | 46 | 20 |
| Vestibularis neuritis | 9 | 3.9 |
| Meniere’s disease | 18 | 7.8 |
| Other | 35 | 15.2 |
| **Non-peripheral vestibular** | 123 | 53 |
| Vertigo of central origin | 21 | 9.1 |
| Other | 102 | 44.1 |
| Diagnosis at the outpatient spine clinic^a^ |  |  |
| Cervicalgia | 97 | 75.2 |
| Tension type headache/migraine | 27 | 21 |
| Myalgia | 22 | 17 |
| Radiculopathy | 4 | 3 |
| BPPV, Benign positional paroxysmal vertigo  ^a^ Some of the patients from the outpatient spine clinic had several  diagnoses | | |
